# Evidence of extensive reef development and high coral cover in nearshore environments: implications for understanding coral adaptation in turbid settings

**DOI:** 10.1038/srep29616

**Published:** 2016-07-19

**Authors:** Kyle M. Morgan, Chris T. Perry, Scott G. Smithers, Jamie A. Johnson, James J. Daniell

**Affiliations:** 1 Department of Geography, College of Life and Environmental Sciences, University of Exeter, Exeter, United Kingdom; 2 College of Science and Engineering, James Cook University, Townsville, Queensland 4811, Australia

## Abstract

Mean coral cover has reportedly declined by over 15% during the last 30 years across the central Great Barrier Reef (GBR). Here, we present new data that documents widespread reef development within the more poorly studied turbid nearshore areas (<10 m depth), and show that coral cover on these reefs averages 38% (twice that reported on mid- and outer-shelf reefs). Of the surveyed seafloor area, 11% had distinct reef or coral community cover. Although the survey area represents a small subset of the nearshore zone (15.5 km^2^), this reef density is comparable to that measured across the wider GBR shelf (9%). We also show that cross-shelf coral cover declines with distance from the coast (R^2^ = 0.596). Identified coral taxa (21 genera) exhibited clear depth-stratification, corresponding closely to light attenuation and seafloor topography, with reefal development restricted to submarine antecedent bedforms. Data from this first assessment of nearshore reef occurrence and ecology measured across meaningful spatial scales suggests that these coral communities may exhibit an unexpected capacity to tolerate documented declines in water quality. Indeed, these shallow-water nearshore reefs may share many characteristics with their deep-water (>30 m) mesophotic equivalents and may have similar potential as refugia from large-scale disturbances.

Coral reefs worldwide are in serious decline, with adverse changes in coral cover^1^,^2^, community structure^3^, habitat structural complexity^4^ and reef-building capacity^5^ occurring in many locations. Climate changes (e.g. elevated sea surface temperature events), outbreaks of coral disease and crown-of-thorns starfish, as well as localised and direct human activities (e.g. overfishing and land-use change)^1^ have been major drivers of this degradation^6^. One such stress derives from the effect of increased sediments from coastal catchments which can smother corals^7^,^8^ and attenuate the penetration of photosynthetically active radiation (PAR) through the water column^9^, with potential negative impacts upon coral growth, reproductive success, and disease susceptibility^10^. Intuitively, sediment-charged waters are therefore commonly perceived as unsuitable (or at best ‘marginal’) for coral reef development. However, recent field investigations^11^,^12^,^13^ have begun to highlight an increasingly diverse range of atypical reef types within Australian waters in which corals appear more resistant to external stressors (e.g. submerged reefs on the central Great Barrier Reef^12^ and nearshore reefs of Bonaparte Archipelago, Western Australia)^13^. These reefs have subsequently formed the basis of modelling studies which suggest that such ‘suboptimal’ reef habitats may act as important future climate-change refugia sites from large-scale disturbance events and may facilitate the natural genetic flow of more stress-resistant corals between reefs^13^,^14^.

Despite the potential significance of such reef-building settings, empirical data on the structure, community composition and diversity of contemporary reefs within nearshore light-limited environments (defined here as “shallow-water mesophotic” reefs, as corals experience similarly low light conditions as deep-water mesophotic coral ecosystems due to very high turbidity) remains sparse. This is largely due to difficult field working conditions especially from poor visibility. Although reef core records have provided clear evidence of prolonged (millennial duration) phases of reef-building under these conditions^15^,^16^,^17^, there is an assumption that contemporary reefs are in poor health. Here, we challenge these perceptions using new seafloor and benthic community data from sites in very nearshore (<10 m isobath) and highly-turbid areas of the central Great Barrier Reef (GBR), Australia. A recent analysis across the wider GBR reports that average coral cover has declined over the past 27 years (from 28% to 13.8% between 1985 and 2012), attributed to major disturbance events (e.g. coral bleaching and tropical cyclones) and outbreaks of crown-of-thorns starfish^6^. Although poorly quantified, similar trajectories are projected for the inner-shelf due to increased terrestrial run-off which has increased five- to ten-fold within the Burdekin river catchment since European settlement^18^,^19^, and which has been reported to have negatively impacted reefs in more distal settings along the inner- and mid-shelf boundary^20^. However, intra-regional variability in the magnitude and types of stresses on corals within inshore reef settings will ultimately influence their ecological health, and those located further away from river mouth outlets^21^ (e.g. Burdekin River), and/or where coastal development is limited, may indeed be able to support coral growth regardless of high sedimentation.

Here, in the largest examination of nearshore coral growth and reef development on the GBR to date, we use data to: 1) define the extent and composition of the nearshore reefs and coral communities within a 15 km^2^ area of central Halifax Bay; 2) establish the antecedent topographic controls on reef development and community structure; and 3) use these parameters to classify patterns of habitat zonation within these turbid environments. Our findings contest conventional views regarding the threshold conditions for coral recruitment and long-term reef-building in nearshore areas. In particular, our results highlight the capacity for naturally marginal marine settings to support productive and diverse reefal habitats, and provide a framework for testing hypotheses about the apparent resilience of these reefs to some of the drivers of coral community degradation associated within seemingly more optimum (high light, lower nutrient) conditions on mid- and outer shelf areas of the central GBR.

## Results

### Spatial extent and relative abundance of nearshore coral communities

**Figure 1:**
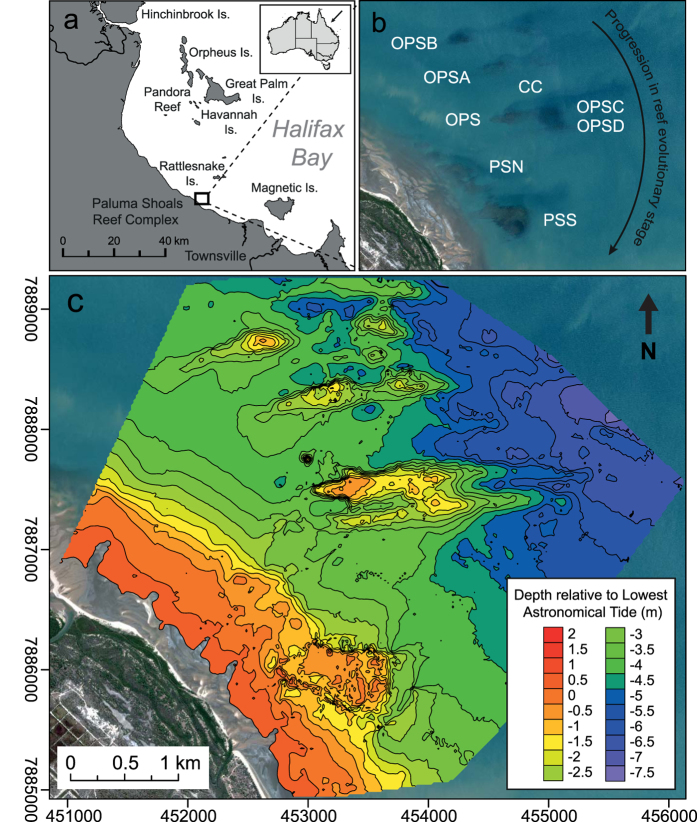
(**a,b**) Paluma Shoals Reef Complex (PSRC) located within Halifax Bay, central Great Barrier Reef, Australia (PS: Paluma Shoals [North & South], OPS: Offshore Paluma Shoals, OPS [A,B,C,D]: Offshore Paluma Shoals [A,B,C,D], and CC: coral carpets). Australian boundaries were imported into ArcMap 10.2.2 (http://www.esri.com/) from the database of Global Administrative Areas (GADM) which is freely available for academic use (http://gadm.org/). (**c**) Seafloor bathymetry (co-ordinates in Australian Map Grid) of the nearshore survey area (15.5 km^2^) generated from single-beam acoustic survey data (contours are at 0.5 m intervals relative to lowest astronomical tide). Contour map was generated in Golden Surfer 12 (http://www.goldensoftware.com/). All maps were modified in Adobe Illustrator Version CS5 (http://www.adobe.com/). WorldView-2 satellite imagery is courtesy of the DigitalGlobe Foundation (http://www.digitalglobefoundation.org/).

**Figure 2:**
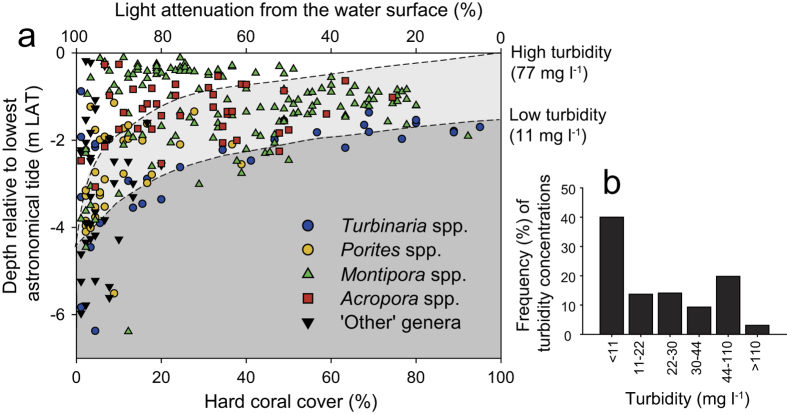
(**a**) Mean relative hard coral cover (%) versus water depth (m below LAT), calculated from 10-frame running averages of benthic cover across Paluma Shoals Reef Complex (PSRC). Symbols denote dominant coral genera at each site. Light attenuation from the water surface (%) is shown for low turbidity (11 mg l^−1^) and high turbidity (77 mg l^−1^) scenarios derived from field measurements by Browne *et al*.^27^ at Paluma Shoals. (**b**) Frequency (%) of turbidity concentrations (mg l^−1^) recorded at Paluma Shoals.

**Figure 3:**
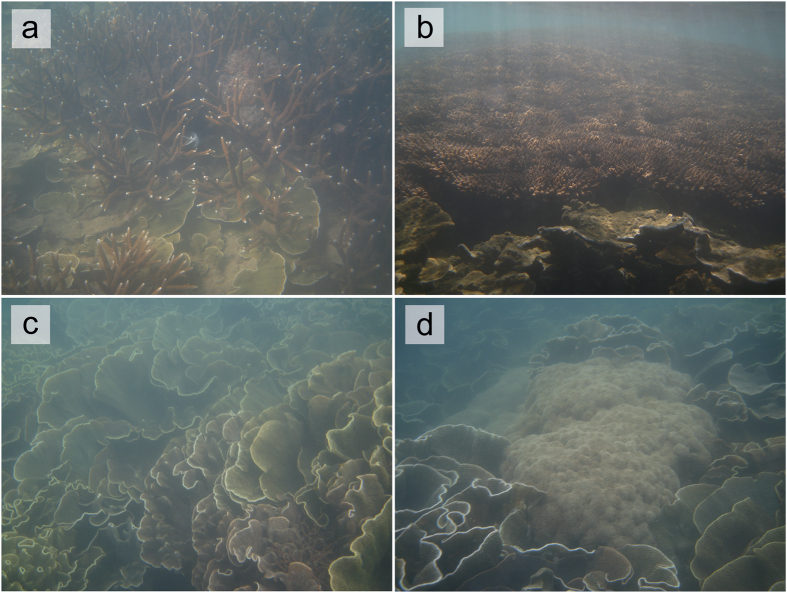
Nearshore coral communities within Paluma Shoals Reef Complex (PSRC): (**a**) shallow water branching *Acropora* spp. and platy *Montipora* spp.; (**b**) tabular *Acropora* spp. (>1 m diameter) common in shallow water areas; (**c**) extensive stands of foliose *Turbinaria* spp. ‘coral carpets’ colonising sandy seafloor areas; (**d**) large *Porites* spp. colony on the sandy seafloor amongst *Turbinaria* spp.

Surveys of the Paluma Shoals Reef Complex (PSRC) encompassed an area of 15.5 km^2^ (Fig. 1), 11% of which was occupied by hard corals. Mean (±s.d.) coral cover within the PSRC was 38 ± 24%, but locally attained coverage of up to 75% across a large area (0.012 km^2^). Highest mean cover (40 ± 36%) was recorded in shallow waters (<2 m below lowest astronomical tide [LAT]), and decreased rapidly with depth to 4 m below LAT, closely following light attenuation under low-turbidity scenarios (11 mg l^−1^; Fig. 2). Below 4 m LAT, reef framework grades into inter-reef sand/muds with very low coral cover (<2%). We also report high structural complexity of reef framework (median rugosity: 3; see Supplementary Table S1), which is a key ecological metric associated with species diversity of reef-dwelling organisms^22^. Despite the limited depth range of living coral, within the PSRC we recorded 21 coral genera (see Supplementary Table S2). Of these taxa, four genera were identified as dominant based on their relative mean abundance: *Montipora* spp. (28 ± 27%), *Acropora* spp. (11 ± 15%), *Turbinaria* spp. (8 ± 17%) and *Porites* spp. (4 ± 8%) (Fig. 3).

**Figure 4:**
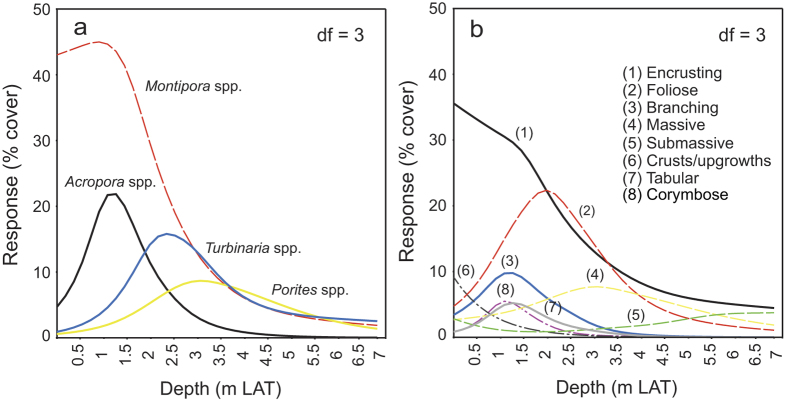
Distribution of response curves of (**a**) common coral genera, and (**b**) coral growth morphotype, with water depth (m below lowest astronomical tide) calculated by a generalised additive model (GAM) with step-wise Akaike Information Criterion (AIC) model selection (df = 3). GAM models were made in CANOCO 5 (http://www.canoco5.com/).

Species response curves generated for the four most common coral genera and all classified coral growth morphologies suggest that corals exhibit clear preferences in their depth distributions (*p* < 0.001; Fig. 4) and are similar to the patterns observed at other nearshore sites where data is available (e.g. Middle Reef)^23^. *Acropora* spp. and *Montipora* spp. dominate shallow water assemblages (<1.5 m LAT; see Supplementary Video S1) and the relative abundance of encrusting taxa (mostly *Montipora* spp.) increases considerably towards sea level, whereas branching and tabular *Acropora* spp. were found to occupy a normally-distributed range between 0.5–2.5 m below LAT (maximum relative cover of 22% at 1 m below LAT). Massive *Porites* spp. and large stands of foliose *Turbinaria* spp. (see Supplementary Video S2) inhabited deeper reefal areas (1.5–4 m LAT and 1.5–3.5 m LAT, respectively). Submassive colonies (e.g. *Lobophyllia* sp., *Galaxea* sp., *Goniopora* sp.) on the seafloor were recorded in very low relative abundances (<5%) at depth (>4 m LAT; Fig. 4).

### Seafloor bathymetry and inter-reef variability in coral assemblages

Comparative analysis of our high-resolution seafloor bathymetry with ecological datasets shows that the main areas of reef development were restricted to a series of transverse antecedent ridge-like structures (Fig. 1c). These influence both the total extent of coral growth and drive significant shifts in community assemblages. Mean (±s.d.) coral cover varied between sites (OPS: 18 ± 26%; OPSA: 22 ± 31%; OPSB: 43 ± 36%; OPSC: 64 ± 30%; OPSD: 53 ± 36%, CC: 71 ± 19%; see Supplementary Table S3). These shore-normal ridges (i.e. running broadly east-west) occur within the <6 m depth zone and are approximately 1–1.7 km long and 200 m wide. It is reasonable to speculate that these ridge structures represent a series of submarine dune ridges (with a sand/mud interior). However, past coring studies^16^,^17^,^24^ clearly demonstrate that most of the topography visible on the seafloor imagery is the result of vertical reef accretion, and thus the elevation of these underlying structures must be assumed to be low (<0.5 m).

We do note a clearly defined north-south gradient in ridge (i.e. reef) topography (Fig. 1b,c), ranging from: (1) shore-attached high elevation reefs with extensive emergent reef flats (Paluma Shoals; +0.5 m LAT); (2) southern reefs, with a continuous double-ridge morphology which form partially-emergent (OPS: 0 m LAT) and fully-submerged structures (OPSC & OPSD: −0.6 m LAT); (3) central reefs, a semi-continuous submerged structure with a mid-ridge depression (OPSA, −0.4 m LAT); and (4) northern reefs, formed of two submerged coalescing structures in early stages of reef development (OPSB, −0.4 m LAT). This diverse topography between reefs at different elevations is indicative of the reefs currently existing at different stages of evolutionary development^25^. In contrast, inter-ridge seafloor areas were relatively flat and featureless, and characterised by sands/muds with sparse coral rubble, sea whips, hydroids, and very low coral cover.

### Habitat zonation of Halifax Bay

**Figure 5:**
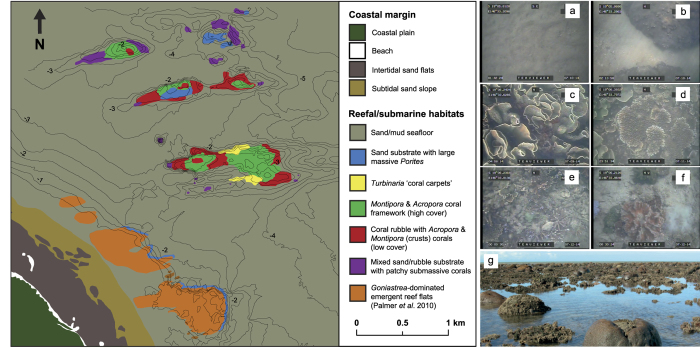
Habitat map of nearshore Halifax Bay, central Great Barrier Reef, Australia. Habitat zones were delineated based on their relative benthic cover and coral community composition in ArcMap 10.2.2 (http://www.esri.com/). Bathymetric contours (at 0.5 m relative to lowest astronomical tide) are shown for topographic reference and were imported from Golden Surfer 12 (http://www.goldensoftware.com/). Ecological datasets for Paluma Shoals North and South were taken from Palmer *et al*.^16^. The final map was modified in Adobe Illustrator Version CS5 (http://www.adobe.com/). Representative images of habitat types are shown: (**a**) sand/mud seafloor; (**b**) sand-dominated substrates with massive Porites rus; (**c**) *Turbinaria mesenterina* “coral carpets”; (**d**) high coral cover reef crests with *Montipora* spp. and *Acropora* spp. framework; (**e**) rubble-dominated with low cover of *Montipora* spp. and *Acropora* spp.; (**f**) sand and rubble with patchy submassive corals (e.g. *Lobophyllia* sp., *Galaxea* sp., *Goniopora* sp.); (**g**) *Goniastrea*-dominated emergent reef flats.

Clustering analysis of benthic substrate types and coral datasets collected from across our study area differentiated six major habitat types with each comprising different benthic associations (Fig. 5; Supplementary Fig. S1). These habitats were: (1) sand-dominated substrates (47 ± 9.2%) with massive *Porites rus* (24 ± 15%) on submerged reef slopes; (2) terrigenous sand/mud (91 ± 13%) covering inter-reef seafloor areas; (3) large *Turbinaria* spp. stands (80 ± 15%), or “coral carpets” (*sensu*)^26^, on the flanks of partially-emergent reefs; (4) high coral cover (47 ± 19%) reef ridges (incipient “crests”) characterised by *Montipora* spp. (55 ± 19%) and *Acropora* spp. framework (16 ± 16%); (5) rubble-dominated (74 ± 12%) emergent reef areas, supporting low coral cover (13 ± 10%) of mainly foliose/encrusting *Montipora* spp. (13 ± 11%) and branching *Acropora* spp. (19 ± 22%); and (6) low coral cover (11 ± 10%) seafloor areas with submassive corals (e.g. *Lobophyllia* sp., *Galaxea* sp., *Goniopora* sp.). A seventh habitat type (*Goniastrea*-dominated emergent reef flats) was identified from existing ecological assessments of Paluma Shoals North (PSN) and South (PSS)^16^ and is included in the final habitat map (Fig. 5). Inter-habitat mean cumulative dissimilarity was 80% (SIMPER analysis; see Supplementary Table S4), indicating that differences in benthic characteristics between the discriminated habitat types were high (i.e. only 20% similar). Soft sediment substrates (sand/mud) identified as the main discriminator for habitat dissimilarity (31%), and the relative abundance of *Montipora* spp. (15%), *Acropora* spp. (6%) and *Turbinaria* spp. (5%) for within coral associations. Between the two coral framework-producing habitats (4 & 5 above), there was a mean cumulative dissimilarity of 55% differentiated by the relative proportion of coral rubble (24%), hard coral cover (20%), and the relative abundance of *Montipora* spp. (24%).

## Discussion

Coral reefs growing within the nearshore zone of the central GBR experience chronic high turbidity as accumulated fine sediments are resuspended by waves^27^,^28^. Because this turbidity rapidly attenuates light availability at depth^9^, these reefs, like their deeper mesophotic cousins, are poorly investigated systems, but it is generally assumed that coral growth and reefal development within these environments is limited by sub-optimal light conditions^10^. Here, however, we document large areas of topographically complex reef composed of relatively diverse (21 genera) and thriving hard coral communities in this shallow, muddy nearshore zone. Importantly, we note the presence of both large (>1 m diameter) long-lived and smaller coral colonies (10–20 cm diameter *Acropora* spp. and *Montipora* spp.) indicating recruitment is occurring within these areas. Previous reef core data from a number of locations along the GBR have shown that reef-building has occurred in isolated nearshore locations throughout the mid- to late Holocene^29^, and extensive stands of foliose *Turbinaria* spp. resembling those at PSRC have been reported elsewhere within a turbid high-latitude setting in Hervey Bay, Queensland, Australia^11^. However, our findings suggest that the scale and diversity of reef-building may be far more extensive than previously thought.

A key implication of this is that these low-light adapted coral communities may be better acclimated to cope with the stresses that have driven the major declines in coral cover observed on mid-outer shelf reefs since the mid-1980’s^6^. Therefore, and as suggested in recent global modelling projections^14^, turbid reef environments that are suitably flushed by tidal currents (such as PSRC) may potentially serve as important refugia sites for corals throughout many of the world’s major reef-building provinces (including eastern Australia), as high suspended sediment concentrations within the water column increase the intensity of light scatter and thus significantly reduce solar irradiance and thermal stress on corals (i.e. the direct effects of ocean warming that cause large-scale coral bleaching). These theoretical scenarios are partially supported by recent reports from the Bonaparte Archipelago, Western Australia, where diverse assemblages (60 genera) of stress-tolerant corals survive extreme environmental conditions, experiencing large fluctuations in sea surface temperature, exposure, turbidity and tidal range^13^, and which have begun to expand our understanding of the tolerance thresholds of reef-building corals in nearshore marginal settings. Indeed, our new data coupled with existing reef core records^16^,^24^ indicate that nearshore areas on the GBR are also capable of sustaining long-term reef development with high rates of vertical reef accretion, which continues to the present until the reefs become sea level constrained. These areas may thus harbour critical but largely-overlooked (and very poorly mapped) habitats for reef communities akin to those deep water (>30 m) mesophotic coral ecosystems that occur along the deeper outer margins of the GBR^30^,^31^.

Specifically, our surveys show high coral cover (mean: 38 ± 24%) within nearshore areas of central Halifax Bay. We note that mean hard coral cover is more than twice that of the average for the central GBR shelf (12%; AIMS long-term reef monitoring; see Supplementary Table S5). Furthermore, our ecological survey data (based on an analysis of 4000+ images from ~50 km of survey lines) indicate that these shallow-water mesophotic reefs support relatively diverse coral assemblages with some 21 coral genera identified within the PSRC, comparable to AIMS studies of inner-shelf (inshore) reefs^32^. Although Cyclone Yasi did disturb some intertidal reef communities in our study area^33^, our data from 2013 and 2014 show that coral assemblages within Halifax Bay were in good condition after the event and that subtidal branching and platy taxa were relatively unaffected. Factors linked to major declines in hard coral cover at a number of GBR mid- and outer-shelf sites^6^, such as partial mortality, disease scars, and crown-of thorns starfish were all largely absent within the study area.

**Figure 6:**
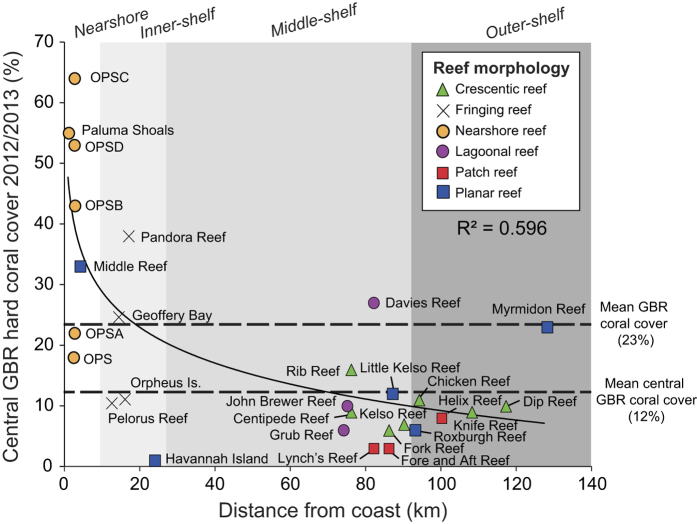
Hard coral cover (%) versus distance from the mainland coast (km) at reef sites on the central Great Barrier Reef (AIMS reef monitoring sector-Townsville), Australia. Hard coral cover estimates (2010–2013) taken from AIMS long-term reef monitoring database (http://data.aims.gov.au/monmap3/cruisereport.jsp?cruise=all). Symbols denote the present reef morphology at each site.

Paradoxically, these nearshore reefs that are proximal to the coast (<3 km) and located relatively close to human-modified catchments, have hard coral cover levels much greater than those measured at many ‘clear-water’ mid- and outer-shelf sites on the central GBR in recent years. Our data shows that mean cover declines rapidly with distance from the mainland coast (R^2^ = 0.596, *F*_1,23_ = 21.1, *p* < 0.001; Fig. 6). Importantly, we also observed no evidence of large-scale *Acropora* die-off within contemporary assemblages at PSRC following European settlement such as that reported at sites ~15 km offshore along the inner-mid-shelf boundary of the central GBR (e.g. Pelorous Island)^20^, and which lie outside these most turbid nearshore areas. Indeed, our surveys show that branched and tabular *Acropora* (*A. pulchra* and *A. hyacinthus*) are common across our sites (mean: 15 ± 17%, but up to 74% in some areas) in depths <2 m LAT (Fig. 3).

A critical aspect to the apparent success of these nearshore corals must therefore lie in their ability to survive the low light conditions associated with high-turbidity. High suspended sediment concentrations, particularly those dark in colour typical of Halifax Bay, significantly reduce PAR to corals^9^. At PSRC light attenuation curves through turbid waters and the influence of local tidal range are clearly major factors driving the relative abundance of corals and in defining the limits of species distribution. We note that coral species known to be well-adapted to the physical and trophic environments of turbid coastal waters (e.g. *Montipora* spp., *Turbinaria* spp. and *Porites* spp.)^34^,^35^ dominated most PSRC reefs. These species typically exhibit greater autotrophic/heterotrophic plasticity as they are able to utilise organic nutrients bound within planktonic organisms and suspended particulates to offset reduced photosynthetic efficiency^7^,^36^, and often display morphotypic adaptations that enhance sediment sloughing and increase light capture (e.g. *Turbinaria mesenterina*)^36^,^37^. This allows corals, such as *Turbinaria* spp., to withstand sediment loads an order of magnitude higher than maximum background conditions^38^ and exploit less desirable habitats to form extensive monospecific stands^11^.

A strong topographic control on coral development within the PSRC is also evident in our data (Fig. 1), and is most likely associated with the physiological requirements of the reef-building corals. Bathymetric surveys showed a number of low-amplitude (1–2 m) submarine antecedent ridges that are attached at the shore and appear rhythmic in distribution, aligned in such a way that suggest they are controlled by longshore currents. These ridges appear to provide sufficient elevation to facilitate initial coral recruitment and, ultimately, reef growth above the soft-sediment seafloor at these sites. This may mitigate the likelihood of corals on the seabed from being smothered during temporary periods of fine material accumulation, and in later stages of vertical reef growth, provide increasing (with shallowing) access to light and repositioning of the corals into the resuspension zone^39^.

Underlying sedimentary structures built by terrigenoclastic sediments thus ironically appear fundamental in determining the location and extent of nearshore reef growth within the PSRC. Reefs rise across a depth range of 0.5 m at the shore-attached Paluma Shoals, to 4 m water depth (OPSB) and occur along a continuum of reef evolutionary states defined by their current position relative to lowest astronomical tide (LAT). These range from deeper incipient coral communities (“coral carpets”), through to juvenile (OPSC & OPSD), late-juvenile (OPSA & OPSB), mature (OPS) and senile reefs (Paluma Shoals), that are fully emplaced at sea level and emergent under low tidal conditions (based on evolutionary classifications of clear-water GBR reefs from)^25^. Reef structures across varying reef evolutionary stages are associated with type-specific coral cover and coral community assemblages, and share many parallels with geological records of Miocene reefs which were also influenced by terrigenoclastic sediment inputs, initiating directly on coarse-grained fluvial fan deposits^40^,^41^. In these early reef settings, reef morphology was similarly controlled by local sedimentary processes rather than independent coral framework growth^42^, as at PSRC.

However, the broader distribution of reefs in the most nearshore areas of the GBR shelf still remains relatively unknown. The widespread cover of hard corals within the mapped area of Halifax Bay suggests that it is highly unlikely that such reef communities are restricted to the PSRC alone. Indeed, preliminary analysis of satellite imagery shows numerous near-surface features (~2 km^2^ of potential reef structure between Townsville and Lucinda) at sites north of the PSRC with similar geometries to those identified here^15^. This indicates that coral reef development may be far more abundant within this coastal nearshore zone than previously thought. However, whether these reefs exhibit comparable coral cover or display similar levels of ecological ‘health’ as PSRC is unknown, as each reef will experience varying degrees of external stress depending on their proximity to major coastal development and/or river mouth outlets. The proportion of seafloor covered by reefal structures within our survey area (11%) is consistent with the average density of reefs on the GBR shelf (9%)^25^, and this thus points to a need to better map and define what has previously been a very over-looked habitat type (both in terms of seafloor topography and the ecological communities which inhabit these areas), and one which in contrast to many mid- and outer-shelf reefs on the GBR have been accreting (and continue to accrete) over contemporary time-scales^24^.

In this context, and based on the relationships between water depth, substrate availability and seafloor topography that we present, we suggest our data could form a useful basis as a predictive model of nearshore reef distribution by assessing suitable habitat extent for coral growth. Under future scenarios of sea-level rise, inundation of low-lying coastal plains will increase accommodation space for coral reef expansion providing new areas for turbid-zone reef growth similar to the reefs that we present here. In turn, understanding reef development within marginal environments is important as they provide close analogues to past and future reefs, and may provide an essential functional role as refugia sites from large-scale disturbance events, or as sources of genetic material (through natural gene flow) of more resistant coral types to reseed degraded reefs.

## Methods

### Study site

Our study focused on the wider Paluma Shoals Reef Complex (PSRC), located within Halifax Bay on the inner-shelf (<3 km from the coast) of the central Great Barrier Reef, Australia (Fig. 1a). Here, several partially-emergent reef structures have previously been identified, two of which have been cored to determine their age and growth history (Paluma Shoals [PS]^15^,^16^; Offshore Paluma Shoals [OPS]^24^; Fig. 1b). In addition, preliminary studies also indicated substantial sub-surface incipient reef and coral cover within proximal seafloor areas of PSRC. We hereafter refer to these discrete reef structures as Offshore Paluma Shoals A, B, C and D (OPS [A, B, C & D]; Fig. 1b). We also make a basic division of the GBR inner-shelf (<20 m isobath at ~20 km offshore)^43^ to differentiate these “coastal nearshore” reefs (<10 m isobath), from other inner-shelf (or “inshore”) reef settings further offshore, which experience different environmental conditions.

The PSRC is located landwards of the shore-detached inshore sediment prism (ISP), a wedge of terrigenoclastic sands that were reworked onshore during the postglacial transgression, and fine material that has accumulated at the shoreline since sea level stabilised in the mid-Quaternary highstand^43^. The PSRC therefore experiences naturally high turbidity (up to 385 mg l^−1^, with 40 days per year exceeding 88 mg l^−128^). As a result these nearshore reefs are extremely light-limited due to sediment laden waters, and thus we classify the PSRC as a series of shallow-water mesophotic reefs. The term “mesophotic” is conventionally used to describe coral ecosystems surviving in deep-water (>30 m) low-light conditions^44^, but was first applied to reefs that experience low-light from muddy waters^45^,^46^. Halifax Bay has a diurnal tidal cycle with a tidal range of 3.6 m.

### Data collection and analysis

We conducted a high-resolution survey of seafloor bathymetry within the PSRC area (15.5 km^2^) to establish the extent and morphology of reefal structures. Because of the shallow nature of the survey area (<8 m depth) bathymetry data was collected using a single-beam echo sounder (Ceeducer Ceestar 200 kHz), coupled with a Real-Time Kinematic Global Positioning System (RTK-GPS). Data were acquired along a suite of closely spaced (every 100 m) north-south parallel lines across the location of known shoals (observed in Landsat imagery), and a series of 500 m spaced shore-perpendicular lines to provide broader context for the shoals (see Supplementary Fig. S2). Depth values were corrected for tidal variations throughout surveying in Caris^TM^ HIPS, and reduced to lowest astronomical tide (LAT) datum using tide gauge data at the Port of Townsville. A digital elevation model of seafloor bathymetry was constructed in Surfer 12 using kriging (10 × 10 m grid size).

Towed video surveys (July 2013/2014) of the seafloor were undertaken using a drop-down video system (SeaViewer with Sea-Track™ GPS overlay), as along with poor visibility due to high turbidity, the presence of saltwater crocodiles and Irukandji jellyfish make long SCUBA transects impractical. The camera was towed 1 m above the seafloor along multiple 300 m transect lines (see Supplementary Fig. S2) to delineate the extent and composition of reefal and non-reefal areas. High-resolution transects (20–40 m spacings) supplemented areas identified as supporting hard coral communities. Still frames (1 m^2^) were extracted from the video at automated 8 sec intervals (*n* = 4420) and a digital 9-point grid overlay was added for analysis of benthic cover. Corals were classified by genera and growth morphology. Reef rugosity within each frame was assessed visually using a modified rugosity classification scheme^47^ (see Supplementary Table S1). Frames were depth-calibrated to LAT datum using the seafloor bathymetric model generated, and 10-frame running averages were used to determine the relative abundance (% cover) of different benthic types. Data collection and analysis produced comparable outputs (% hard coral cover/% coral genera) to those reported by the Australian Institute of Marine Science (AIMS) allowing us to directly compare our findings to AIMS survey data collected at a range of sites within the central GBR region (sector-Townsville, 2010–2013). A habitat map of the nearshore Halifax Bay study area was constructed by combining spatially-corrected benthic cover and bathymetry thematic layers with multi-spectral satellite imagery (WorldView-2 courtesy of DigitalGlobe Foundation) in ArcGIS 10.2.2.

### Habitat classification and statistical analysis

Benthic data was classified into different habitat types using hierarchical agglomerative clustering with group-average sorting^48^,^49^. Non-transformed data, allowing for dominant species or substrata to exert an appropriate influence on habitat cluster groups^49^,^50^, were used to construct a Bray-Curtis similarity matrix from which nearshore reef habitats were identified (<70% similarity). Habitats were distinguished based on their relative cover of substrate type (e.g. sand, rubble, etc.) and coral genera. A similarity percentage (SIMPER) analysis was run to establish biota or substrata driving inter-habitat (dis)similarity^48^,^49^. Species responses (coral genera and growth morphology) to depth (LAT) were examined using a generalised additive model (GAM) generated in CANOCO 5 by applying a Poisson distribution (df = 3), as it provides greater flexibility to fit non-linear relationships. Model selection was determined by a step-wise Akaike Information Criterion (AIC). Regression analyses were conducted to test for differences in coral cover with water depth, as well as cross-shelf changes in mean coral cover with distance from the coast.

## Additional Information

**How to cite this article**: Morgan, K. M. *et al*. Evidence of extensive reef development and high coral cover in nearshore environments: implications for understanding coral adaptation in turbid settings. *Sci. Rep.* **6**, 29616; doi: 10.1038/srep29616 (2016).

## Supplementary Material

Supplementary Video S1

Supplementary Video S2

Supplementary Information
