# Supplementary material for: Evidence of extensive reef development and high coral cover in nearshore environments: implications for understanding coral adaptation in turbid settings
Source: Sci Rep. 2016 Jul 19;6:29616. doi: 10.1038/srep29616 (PMC4949480; doi:10.1038/srep29616)
Supplement: Supplementary Information [file srep29616-s3.pdf]

Evidence of extensive reef development and high coral cover in nearshore environments: implications for understanding coral adaptation in turbid settings

K. M. Morgan, C.T. Perry, S.G. Smithers, J. A. Johnson and J. J. Daniell

Supplementary Information

Supplementary Table S1. Reef rugosity classification scheme (1-5), modified from Polunin and Roberts (1993), used to categorise the three-dimensionality of reef benthic communities and substrata within Paluma Shoals Reef Complex (PSRC).

Supplementary Table S2. Inventory of coral genera recorded within Paluma Shoals Reef Complex (PSRC).

Supplementary Table S3. Mean (s.d.) relative abundance (%) of hard coral assemblages between reef sites surveyed within Paluma Shoals Reef Complex (PSRC).

Supplementary Table S4. SIMPER analysis of pooled habitat groups showing discriminating substrata or coral genera driving average dissimilarity between habitat classifications at Paluma Shoals Reef Complex (PSRC). Mean relative abundance (%) of benthic cover for each of the six habitat classifications is presented.

Supplementary Table S5. Cross-shelf hard coral cover (%), distance from the coast and reef morphology for reef sites within the central Great Barrier Reef (sector-Townsville) reported by Australian Institute of Marine Science (AIMS) long-term reef monitoring programme (http://data.aims.gov.au/monmap3/cruisereport.jsp?cruise=all). Values from the present study at Paluma Shoals Reef Complex (PSRC) are included.

Supplementary Figure S1. Dendrogram of 442 sites within Paluma Shoals Reef Complex (PSRC) using group average clustering from Bray-Curtis similarities. Six habitat cluster groups which are <70% similar were classified from benthic cover (%) data of substrata type and coral genera.

Supplementary Figure S2. Seafloor mapping of Paluma Shoals Reef Complex (PSRC) showing the position of single-beam survey lines used for the construction of the bathymetric model (black lines) and location of still frame sites (10-frame running average) for the analysis of benthic cover (yellow circles). Map was generated in ArcMap 10.2.2 (http://www.esri.com/) using WorldView-2 satellite imagery courtesy of the DigitalGlobe Foundation (http://www.digitalglobefoundation.org/) as a basemap.

Supplementary Video S1. High coral cover and reef rugosity within nearshore coral communities of Halifax Bay, central Great Barrier Reef, Australia. Reef communities are typically dominated by *Montipora* spp. and *Acropora* spp. corals.

Supplementary Video S2. Extensive stands of foliose coral framework (mostly *Turbinaria* spp.) within nearshore Halifax Bay.

Supplementary Table S1. Reef rugosity classification scheme (1-5), modified from Polunin and Roberts (1993), used to categorise the three-dimensionality of reef benthic communities and substrata within Paluma Shoals Reef Complex (PSRC).

Scale (1-5)	Description	Representative images	
1	Flat and featureless, typically soft-sediment sand/muds. No three-dimensionality.	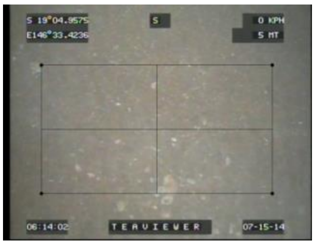	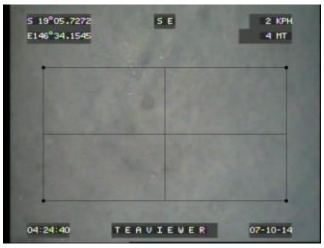
2	Low rugosity coarse gravel/rubble with isolated dead or live coral framework	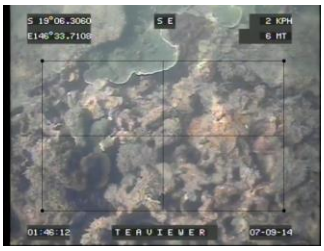	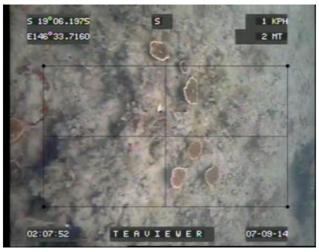
3	Moderate rugosity, high coral cover comprising typically low-profile colonies (e.g encrusting or tabular).	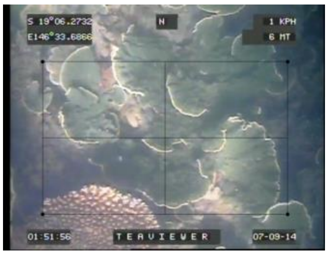	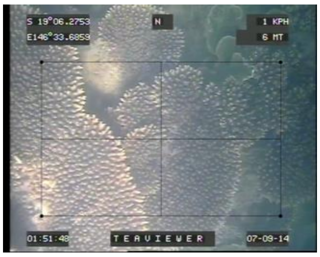
4	High rugosity, mixed structurally-complex coral framwork (branching, tabular and foliose).	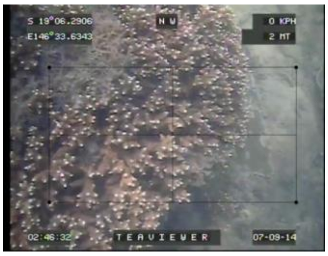	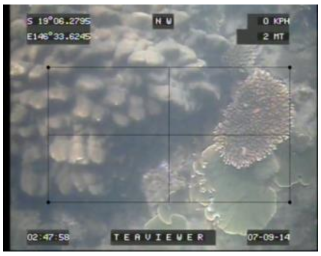
5	Very high rugosity (associated with high coral cover), high three-dimensionality with many crevices and overhangs.	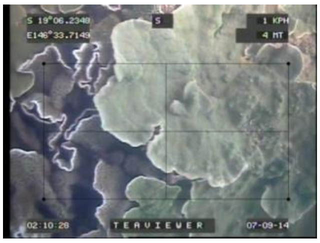	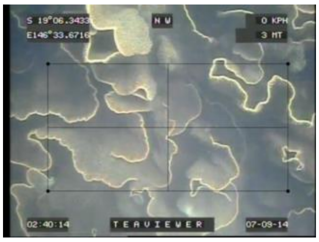

Supplementary Table S2. Inventory of coral genera recorded within Paluma Shoals Reef Complex (PSRC).

Coral genera	
Acropora	*Hydnophora*
Turbinaria	*Platygyra*
Montipora	*Favities*
Porites	*Favia*
Goniastrea	*Pocillopora*
Fungia	*Galaxea*
Stylophora	*Symphyllia*
Cyphastrea	*Euphyllia*
Pectinia	*Pavona*
Lobophyllia	*Caulastrea*
Goniopora	

Supplementary Table S3. Mean (s.d.) relative abundance (%) of hard coral assemblages between reef sites surveyed within Paluma Shoals Reef Complex (PSRC).

	OPS	OPSA	OPSB	OPSC	OPSD	CC
Depth at highest point (m LAT)	0	-0.4	-0.4	-0.6	-0.6	-0.4
Hard coral (%)	18 (26)	23 (31)	44 (37)	64 (30)	54 (36)	72 (20)
Acropora spp. (%)	10 (28)	30 (41)	32 (38)	10 (21)	26 (33)	3 (3)
Montipora spp. (%)	4 (18)	8 (23)	1 (10)	6 (18)	21 (30)	80 (15)
Turbinaria spp. (%)	71 (42)	38 (43)	63 (40)	81 (28)	31 (31)	5 (6)
Porites spp. (%)	3 (15)	17 (36)	3 (14)	1 (6)	16 (36)	1 (3)
Other coral (%)	11 (28)	6 (22)	1 (7)	1 (8)	6 (20)	1 (3)
Reef rugosity (R)	1.2 (0.6)	2.8 (0.8)	3.1 (0.7)	3.8 (1)	4.2 (0.9)	5 (0.5)

Supplementary Table S4. SIMPER analysis of pooled habitat groups showing discriminating substrata or coral genera driving average dissimilarity between habitat classifications at Paluma Shoals Reef Complex (PSRC). Mean relative abundance (%) of benthic cover for each of the six habitat classifications is presented.

	Average dissimilarity	Contribution (%)	Cumulative (%)	Mean relative abundance (%)					
				Habitat 1	Habitat 2	Habitat 3	Habitat 4	Habitat 5	Habitat 6
Sand	25.5	31.6	31.6	46.8	91.2	18.3	3.0	3.3	28.7
Rubble	14.0	17.4	49.0	17.6	4.4	5.6	32.5	74.0	54.6
Montipora	12.5	15.5	64.5	13.5	1.6	5.1	54.9	13.0	8.4
Live coral	11.7	14.5	79.0	21.4	1.9	72.1	46.8	13.1	11.0
Acropora	5.1	6.3	85.3	2.3	0.2	3.2	15.7	19.3	6.5
Turbinaria	3.9	4.8	90.1	2.1	1.3	79.5	7.2	2.7	3.0
Dead coral	3.1	3.9	94.0	8.5	1.0	4.2	11.5	5.9	3.5
Porites	2.0	2.4	96.4	25.5	1.7	1.1	1.8	2.3	7.5
Other coral	1.7	2.1	98.5	3.1	1.6	1.2	3.4	3.1	7.5
Macroalgae	1.2	1.5	100.0	0.0	0.2	0.0	4.1	2.5	0.4
Mean average dissimilarity: **80.62**									

Supplementary Table S5. Cross-shelf hard coral cover (%), distance from the coast and reef morphology for reef sites within the central Great Barrier Reef (sector-Townsville) reported by Australian Institute of Marine Science (AIMS) long-term reef monitoring programme (http://data.aims.gov.au/monmap3/cruisereport.jsp?cruise=all). Values from the present study at Paluma Shoals Reef Complex (PSRC) are included.

Reef	Shelf location	Mean hard coral (%)	Distance from coast (km)	Year	AIMS site number	Reef type
Paluma Shoals	Nearshore	55	1.1	2010	Palmer et al. 2010	Marginal
OPS	Nearshore	18	2.5	2013	This study	Marginal
OPSD	Nearshore	53	2.7	2014	This study	Marginal
OPSC	Nearshore	64	2.7	2014	This study	Marginal
OPSA	Nearshore	22	2.7	2013	This study	Marginal
OPSB	Nearshore	43	2.8	2013	This study	Marginal
Middle Reef	Nearshore	33	4.2	2013	(No. 19-011)	Planar reef
Geoffery Bay	Inner	25	11	2011	No description	Fringing reef
Pelorus	Inner	9	14	2011	No description	Fringing reef
Orpheus Island East	Inner	10	17	2010	No description	Fringing reef
Pandora Reef	Inner	38	17	2013	(No. 18-065)	Fringing reef
Havannah Island	Inner	1	24	2013	(No. 18-051)	Planar reef
Grub Reef	Middle	6	74	2012	(No. 18-077)	Lagoonal reef
John Brewer Reef	Middle	10	75	2013	(No. 18-075)	Lagoonal reef
Centipede Reef	Middle	9	76	2012	(No. 18-088)	Crescentic reef
Rib Reef	Middle	16	76	2013	(No. 18-032)	Crescentic reef
Davies Reef	Middle	27	82	2013	(No. 18-096)	Lagoonal reef
Lynch's Reef	Middle	3	82	2012	(No. 18-091)	Patch reef
Fork Reef	Outer	6	86	2012	(No. 18-083)	Crescentic reef
Fore and Aft Reef	Middle	3	86	2012	(No. 18-043)	Patch reef
Little Kelso Reef	Middle	12	87	2012	(No. 18-031)	Planar reef
Kelso Reef	Middle	7	90	2012	(No. 18-030)	Crescentic reef
Roxburgh Reef	Middle	6	93	2012	No description	Planar reef
Chicken Reef	Outer	11	94	2013	(No. 18-086)	Crescentic reef
Helix Reef	Middle	8	100	2012	(No. 18-076)	Patch reef
Knife Reef	Outer	9	108	2012	(No. 18-081)	Crescentic reef
Dip Reef	Outer	10	117	2013	(No. 18-039)	Crescentic reef
Myrmidon Reef	Outer	23	128	2013	(No. 18-034)	Planar reef

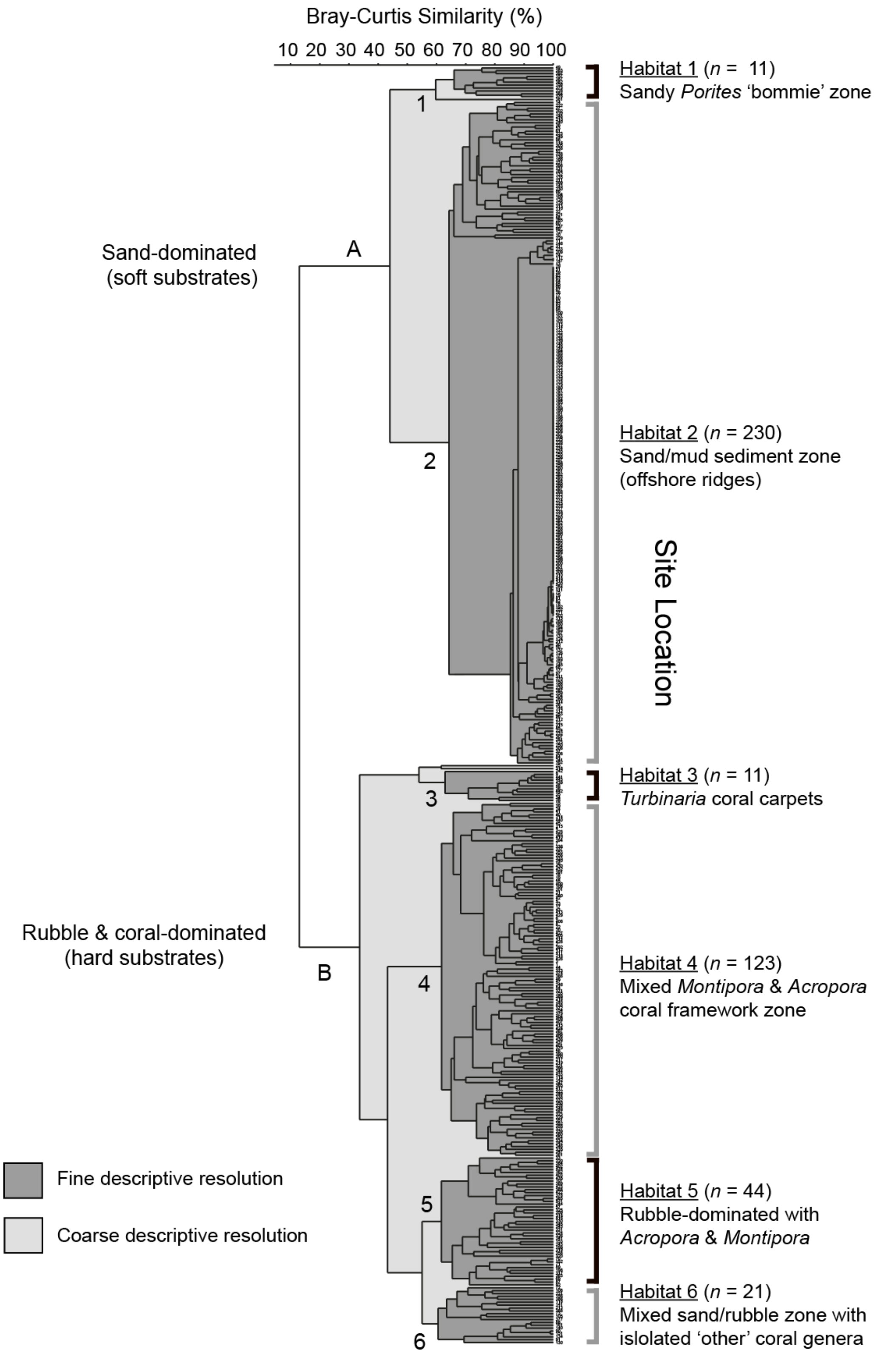

Supplementary Figure S1. Dendrogram of 442 sites within Paluma Shoals Reef Complex (PSRC) using group average clustering from Bray-Curtis similarities. Six habitat cluster groups which are <70% similar were classified from benthic cover (%) data of substrata type and coral genera.

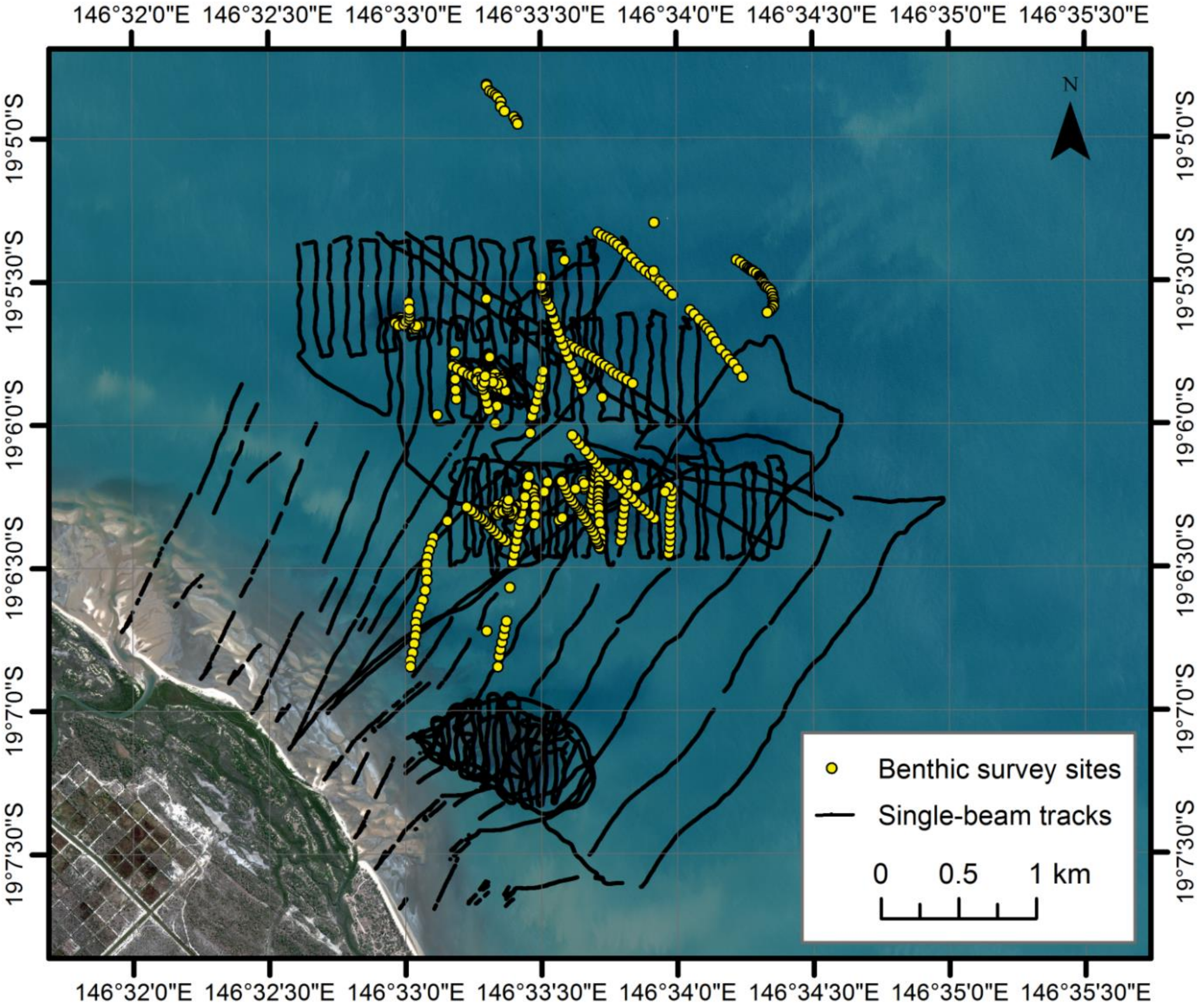

Supplementary Figure S2. Seafloor mapping of Paluma Shoals Reef Complex (PSRC) showing the position of single-beam survey lines used for the construction of the bathymetric model (black lines) and location of still frame sites (10-frame running average) for the analysis of benthic cover (yellow circles). Map was generated in ArcMap 10.2.2 (http://www.esri.com/) using WorldView-2 satellite imagery courtesy of the DigitalGlobe Foundation (http://www.digitalglobefoundation.org/) as a basemap.
